# Efficacy of transcatheter arterial embolization for first-line treatment of colonic diverticular bleeding with extravasation on contrast-enhanced computed tomography

**DOI:** 10.1097/MD.0000000000031442

**Published:** 2022-11-04

**Authors:** Yuki Kojima, Takahito Katano, Takaya Shimura, Masashi Shimohira, Tomoya Sugiyama, Masahide Ebi, Takahito Harada, Yuki Yamamoto, Yoshikazu Hirata, Hiromi Kataoka

**Affiliations:** a Department of Gastroenterology and Metabolism, Nagoya City University Graduate School of Medical Sciences, Kawasumi, Mizuho-cho, Mizuho-ku, Nagoya, Japan; b Department of Radiology, Nagoya City University Graduate School of Medical Sciences, Kawasumi, Mizuho-cho, Mizuho-ku, Nagoya, Japan; c Department of Gastroenterology, Aichi Medical University School of Medicine, Yazakokarimata, Nagakute, Aichi, Japan; d Department of Gastroenterology, Kasugai Municipal Hospital, Takaki-cho, Kasugai, Aichi, Japan.

**Keywords:** colonic diverticular bleeding, endoscopic hemostasis, rebleeding, transcatheter arterial embolization

## Abstract

Colonic diverticular bleeding (CDB) is the most frequent cause of acute lower gastrointestinal bleeding. The aim of this study was to evaluate the efficacy and safety of transcatheter arterial embolization (TAE) for CDB as first-line treatment with extravasation on contrast-enhanced computed tomography (CECT), compared with endoscopic hemostasis. Three Japanese institutions participated in this retrospective cohort study. Data from consecutive patients admitted with a diagnosis of CDB with extravasation on CECT were reviewed. One hospital performed TAE and the others conducted urgent colonoscopy (CS) as the first-line treatment for CDB with extravasation on CECT. The primary outcome was rebleeding rate within 30 days after first-line treatment. In total, 165 CDB cases with extravasation on CECT (TAE group, n = 39; CS group, n = 126) were analyzed in this study. The rebleeding rate within 30 days was significantly lower in the TAE group (7.69%) than in the CS group (23.02%; *P* = .038). The bleeding point detection rate was significantly higher in the TAE group (89.74%, 35/39) than in the CS group (37.30%, 47/126; *P* < .0001). Even in those cases in which a bleeding point was detected, the rebleeding rate was significantly lower in the TAE group (0%) than in the endoscopic hemostasis-success group (23.91%; *P* = .005). No severe complications of Grade 3 or more were seen with TAE. We showed that TAE is an effective, safe hemostatic method, and a useful alternative to endoscopic hemostasis for first-line treatment of CDB.

## 1. Introduction

Acute lower gastrointestinal bleeding (LGIB) is one of the most common clinical emergencies and represents a frequent cause of acute hospital admissions. Colonic diverticular bleeding (CDB) is the most frequent cause of LGIB, accounting for 20% to 48% of all LGIB cases.^[1]^ Patients typically present with massive, painless hematochezia. The incidence of CDB is increasing due to the aging of the population and the increasing use of non-steroidal anti-inflammatory drugs and anticoagulants for age-related arteriosclerosis.^[2–4]^ CDB shows spontaneous hemostasis in 66% to 92.4% of cases, but recurrent bleeding or fatal massive bleeding sometimes occurs.^[5,6]^

Colonoscopy is performed as the initial diagnostic modality for identifying the source of LGIB, including CDB. Previous studies have suggested that colonoscopy should be performed within 24 hours of a hospital visit to maximize the identification rate for stigmata of recent hemorrhage (SRH). According to previous studies, the accuracy of identifying a bleeding source is 26.4% in colonoscopy.^[7]^

With technological advances in medical devices, contrast-enhanced computed tomography (CECT) is increasingly useful for diagnosing bleeding sites in acute LGIB. With CECT, the accuracy of identifying a bleeding site has been reported as 88.2%, higher than the accuracy of colonoscopy.^[8]^ CECT also improves the rate of achieving endoscopic hemostasis for CDB patients by improving the rate of identifying bleeding sources during colonoscopy. Our group has already demonstrated that the identification rate for bleeding diverticulum was higher on colonoscopy in CDB patients showing extravasation of contrast agent on CECT (60.0%) than in CDB patients without extravasation on CECT (31.4%).^[9]^ According to previous studies, the rate of detecting bleeding diverticulum on colonoscopy increased to 60% to 70% in CDB patients showing extravasation on CECT.^[9–11]^

The benefit of colonoscopy within 24 hours in CDB is to improve the SRH identification rate. Colonoscopy after CECT improves both the SRH identification rate and the endoscopic treatment achievement rate. However, whether endoscopic homeostasis prevents rebleeding in CDB is still controversial. A randomized controlled trial revealed that the 30-day rebleeding rate did not differ between patients treated with endoscopic hemostasis and those treated without endoscopic hemostasis.^[12]^ Particularly in CDB patients showing extravasation on CECT, the bleeding site has already been identified. For such patients, subsequent colonoscopy after CECT may not be beneficial and an alternative strategy for CDB treatment seems warranted. However, no studies have evaluated the efficacy of hemostatic methods to reduce the rebleeding rate among CDB patients displaying extravasation on CECT. Transcatheter arterial embolization (TAE) for CDB is a therapeutic option in cases where endoscopic hemostasis fails or bleeding continues to recur. TAE is also considered the treatment of choice for CDB patients with poor systemic conditions. The technical success rate of TAE for LGIB is high, at 93% to 100%, and the rebleeding rate after TAE for CDB is low, at 13% to 14.3%.^[13–15]^ TAE may thus offer a useful alternative method for achieving hemostasis in CDB with extravasation on CECT.

Considering the reported results, our institute has been performing TAE as the first-line treatment for CDB showing extravasation on CECT. To date, no reports have confirmed the efficacy of TAE for the first-line treatment of CDB showing extravasation on CECT. We therefore conducted this retrospective study to elucidate the efficacy of TAE as the first-line treatment to reduce rebleeding in CDB patients with extravasation on CECT (TAE group), as compared with colonoscopy (CS group).

## 2. Materials and methods

### 2.1. Study design and setting

**Figure 1.**
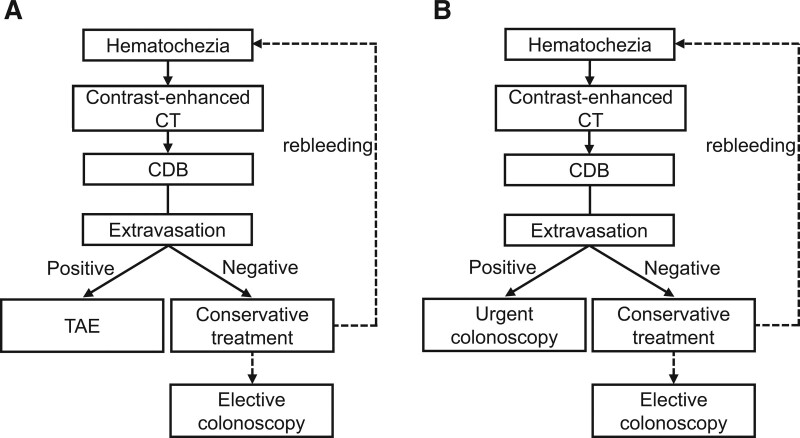
First-line treatment strategy for CDB in the participating institutions. (A) Strategy at NCU. (B) Strategy at AMU and K hospital. AMU = Aichi Medical University hospital, CDB = colonic diverticular bleeding, K hospital = Kasugai Municipal Hospital, NCU = Nagoya City University Hospital.

This retrospective cohort study used data from CDB patients collected from Nagoya City University Hospital (NCU), Aichi Medical University Hospital (AMU) and Kasugai Municipal Hospital (K hospital). NCU and AMU are university hospitals in Aichi, Japan, while K hospital is a tertiary-care center that covers a population of approximately 300,000 people. In NCU, TAE has been performed as the standard first-line treatment for CDB with extravasation on CECT since 2010 (Fig. 1A). Therefore, in NCU, consecutive patients with CDB treated between June 2010 and July 2021 were retrospectively collected. On the other hand, in AMU and K hospital, colonoscopy has been performed as the standard first-line treatment for CDB with extravasation on CECT only since 2016. As a result, in both hospitals, consecutive patients with CDB treated between June 2016 and July 2021 were collected (Fig. 1B).

### 2.2. Study participants

Participants ≥20 years old and admitted to NCU, AMU or K hospital with a diagnosis of CDB were enrolled in this study. The diagnosis of CDB in the database was based on the following criteria: detection of extravasation from a diverticulum on CECT; identification of a colonic diverticulum found with SRH on colonoscopy; or no findings of bleeding source other than CDB by CT or colonoscopy. In participating institutions, CECT is the initial diagnostic modality for identifying the bleeding source in patients with presumed CDB. However, CECT was not able to be performed in patients with renal failure, asthma, and/or allergy to contrast media. In addition, CECT was not performed in patients for a small amount of bleeding with stable vital signs or a long time from the last hematochezia. We excluded the following patients: patients who did not undergo CECT; patients who received conservative treatment for CDB; or patients who received colonoscopy as the first-line treatment in the NCU because of bias in treatment selection.

### 2.3. Outcomes

The primary outcome in this study was the rebleeding rate within 30 days after first-line treatment. Rebleeding was defined as a significantly large amount of fresh bloody or dark red stool along with any of the following: hemodynamic instability; a need for transfusion; identification of blood pooling or SRH in the colonic diverticulum on colonoscopy; or detection of extravasation on CECT. We also analyzed procedure time, hospital stay, units of blood transfused during hospitalization, need for surgery, the rate of bleeding point detection on angiography or colonoscopy, and the treatment achievement rate of TAE or endoscopic hemostasis. Adverse events (AEs) were graded according to Common Terminology Criteria for Adverse Events, version 5.0.^[16]^

### 2.4. Therapies for CDB

In this study, TAE was the first-line treatment for CDB at NCU. CS was the first-line treatment for CDB at AMU and K hospital. At NCU, CS was performed as the first-line treatment in case of shortage of radiological manpower.

In the TAE group, all angiographies were performed by interventional radiology specialists in the Department of Radiology at NCU. TAE was immediately attempted in all patients when extravascular leakage of contrast agents was identified. At NCU, TAE is performed by super-selective catheterization of the vasa recta and embolization using microcoils. If super-selective catheterization was impossible, embolization was performed using embolic agents instead of microcoils.

In the CS group, endoscopic hemostasis was attempted in all CDB patients with identifiable SRH. Endoscopic hemostasis for CDB was performed with monotherapy using endoscopic clipping. Endoscopic clips were placed directly onto the vessel if technically feasible (direct clipping). When direct placement of endoscopic clips onto the vessel was not possible, the diverticulum was closed in a zipper fashion (indirect clipping).

### 2.5. Statistical analysis

Data were analyzed using the chi-square test, Fisher’s exact test, and Mann–Whitney *U* test, as appropriate. Values of *P* < .05 were considered statistically significant. All statistical analyses were performed using JAMP software version 14 (SAS Institute, Cary, NC).

### 2.6. Ethics statement

The present study complied with the STROBE statement.^[17]^ The ethics committees and institutional review boards of all participating hospitals approved performance of this study with the opt-out method (The ethics committee of Nagoya City University: Institutional Review Board no. 2021-155; The ethics committee of Aichi Medical University: Institutional Review Board no. 2021-542; The ethics committee of Kasugai Municipal Hospital: Institutional Review Board no. 496). The requirement for obtaining informed consent from patients before study participation was waived, as no patients underwent additional interventions, the study was retrospective and observational in nature, and the data were collected anonymously. The study was conducted according to the ethical guidelines of the 1975 Declaration of Helsinki (7th revision, 2013).

## 3. Results

### 3.1. Patient selection

**Figure 2.**
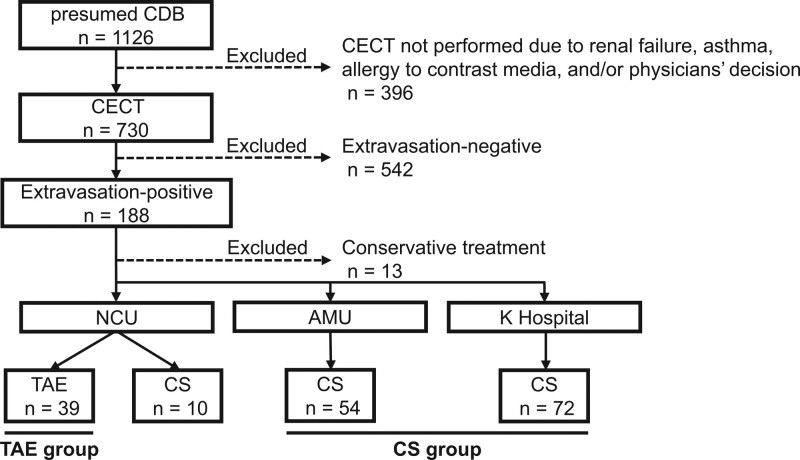
Study flowchart. The database recorded 1126 CDB cases, of which we excluded 961 cases meeting the exclusion criteria (396 cases without CECT on admission; 542 cases without extravasation on CECT; 13 cases with conservative treatment; 10 cases with colonoscopy for first-line treatment in NCU). Finally, 165 CDB cases with extravasation on CECT were analyzed in this study, comprising 39 in the TAE group and 126 cases in the CS group. CDB = colonic diverticular bleeding, CECT = contrast-enhanced computed tomography, CS = colonoscopy, NCU = Nagoya City University Hospital, TAE = transcatheter arterial embolization.

The database recorded 1126 CDB cases, of which we excluded 961 cases meeting exclusion criteria (396 cases without CECT on admission; 542 cases without extravasation on CECT; 13 cases with conservative treatment; and 10 cases with colonoscopy as first-line treatment in NCU) (Fig. 2). Finally, 165 CDB cases with extravasation on CECT were analyzed in this study, with 39 cases in the TAE group and 126 cases in the CS group. No CDB patients received TAE as first-line treatment at AMU and K hospital, or surgery as first-line treatment at any of the institutions during the study period. In the TAE group, interventional radiology was performed within 24 hours after visit in all 32 cases. In the CS group, colonoscopy was performed within 24 hours after visit in 119 cases, within 24 to 48 hours in 6 cases, and within 48 to 72 hour in 1 case.

### 3.2. Patient characteristics

Characteristics of the TAE group (n = 39) and CS group (n = 126) are shown in Table 1. A significantly greater proportion of patients in the TAE group had higher systolic blood pressure <100 mm Hg and hemoglobin <10 g/dL compared to the CS group (15.8% vs 1.59% and 43.6% vs 20.6%, respectively; *P* = .002 and *P* = .004, respectively).

**Table 1:** Background characteristics of patients.

	TAE group (n = 39)	CS group (n = 126)	*P*
Age (yr)	71 [66–79]	76 [69–81]	.197^‡^
Sex, male	29 (74.4%)	84 (66.7%)	.366^*^
BMI, kg/m^2^	23.2 [20.9–26.1]	22.3 [19.9–25.4]	.204^‡^
History of diverticular bleeding	13 (33.3%)	63 (50.0%)	.068^*^
Comorbidity			
Hypertension	25 (64.1%)	76 (60.3%)	.672^*^
Diabetes mellitus	10 (25.6%)	28 (22.2%)	.658^*^
Ischemic heart disease	10 (25.6%)	27 (21.4%)	.582^*^
Cerebrovascular disease	7 (18.0%)	21 (16.7%)	.852^*^
Chronic kidney disease	4 (10.3%)	15 (11.9%)	1.0^†^
Atrial fibrillation	3 (7.7%)	20 (15.9%)	.290^†^
Medication			
Anti-platelet	13 (33.3%)	49 (38.9%)	.531^*^
Anticoagulant	4 (10.3%)	19 (15.1%)	.600^†^
Antithrombotic drugs ≥ 2	5 (12.8%)	15 (11.9%)	1.0^†^
NSAIDs	8 (20.5%)	16 (12.7%)	.226^*^
Time from first hematochezia to CECT	4.6 [2.5–8]	4 [2.5–8]	.668^‡^
SBP < 100 mm Hg	6 (15.8%)	2 (1.59%)	**.002** ^†^
HR > 100 beats/min	10 (26.3%)	46 (36.5%)	.246^*^
Hb < 10 g/dL	17 (43.6%)	26 (20.6%)	**.004** ^*^
Bleeding diverticulum site			.300^*^
Cecum	4 (10.3%)	5 (4.0%)	
Ascending colon	24 (61.5%)	74 (58.7%)	
Transverse colon	3 (7.7%)	5 (4.0%)	
Descending colon	3 (7.7%)	20 (15.9%)	
Sigmoid colon	5 (12.8%)	22 (17.5%)	

Values are presented as number (%) or median [IQR].

Bold values denote statistical significance at the *P* < .05 level.

BMI = body mass index, CECT = contrast-enhanced computed tomography, CS = colonoscopy, Hb = hemoglobin, HR = heart rate, IQR = interquartile range, NSAIDs = non-steroidal anti-inflammatory drugs, SBP = systolic blood pressure, TAE = transcatheter arterial embolization.

* Chi-square test.

† Fisher’s exact test.

‡ Mann–Whitney *U* test.

### 3.3. Main analysis

Rebleeding within 30 days after first-line treatment occurred in 3 of 39 cases in the TAE group and 29 of 126 in the CS group. The rebleeding rate within 30 days was significantly lower in the TAE group (7.69%) than in the CS group (23.02%; *P* = .038) (Table 2). Median procedure time of TAE was 68 min, significantly longer than that of CS (42 minutes; *P* < .001). Hospital stay was significantly longer in the TAE group (11 days) than in the CS group (8 days; *P* = .008). Significantly more blood units were transfused in the TAE group (4 units) than in the CS group (0 units; *P* < .001). The need for surgery did not differ significantly among the TAE group (2.56%) and CS group (2.38%; *P* = 1.0). The rate of bleeding point detection was significantly higher in the TAE group (89.74%) than in the CS group (37.30%; *P* < .0001).

**Table 2:** Comparison of clinical outcomes between TAE group and CS group.

	TAE group (n = 39)	CS group (n = 126)	*P*
Rebleeding rate within 30 d	3 (7.69%)	29 (23.02%)	**.038** ^†^
Procedure time (min)	68 [44–85]	42 [30–58]	**<.001** ^‡^
Hospital stay (d)	11 [7–14]	8 [6–11]	**.008** ^‡^
Blood transfusion units	4 [0–6]	0 [0–2]	**<.001** ^‡^
Need for surgery	1 (2.56%)	3 (2.38%)	1.0^†^
Bleeding point detection rate	35 (89.74%)	47 (37.30%)	**<.001** ^*^

Values are presented as number (%) or median [IQR].

Bold values denote statistical significance at the *P* < .05 level.

CS = colonoscopy, IQR = interquartile range, TAE = transcatheter arterial embolization.

* Chi-square test.

† Fisher’s exact test.

‡ Mann–Whitney *U* test.

### 3.4. Subgroup analysis

Next, we analyzed the impact of successful treatment on clinical outcomes. In the TAE group, 29 of 39 CDB cases received successful embolization of the bleeding artery (TAE-success) and 10 of 39 were angiography alone at initial treatment (TAE-failure). Rebleeding rate within 30 days was lower with TAE-success (0%) than with TAE failure (30%; *P* = .013) (Table 3). No significant differences in hospital stay, units of blood transfused or need for surgery were evident between TAE-success and TAE-failure.

**Table 3:** Comparison of clinical outcomes between TAE-success and TAE-failure.

	TAE-success (n = 29)	TAE-failure (n = 10)	*P*
Rebleeding rate within 30 d	0 (0%)	3 (30%)	**.013** ^*^
Hospital stay (d)	14 [7–20]	10 [8–13]	.333^†^
Blood transfusion units	4 [0–6]	0 [0–7]	.566^†^
Need for surgery	0 (0%)	1 (10%)	.256^*^

Values are presented as number (%) or median [IQR].

Bold values denote statistical significance at the *P* < .05 level.

IQR = interquartile range, TAE = transcatheter arterial embolization.

* Fisher’s exact test.

† Mann–Whitney *U* test.

In the CS group, 46 of 126 cases achieved endoscopic clipping hemostasis at initial treatment (EH-success) and 80 of 126 cases did not achieve endoscopic hemostasis (EH-failure). No significant differences in rebleeding rate within 30 days, hospital stay, units of blood transfused or need for surgery were seen between EH-success and EH-failure (Table 4). In EH-success group, direct clipping and indirect clipping were performed in 14 and 32 cases, respectively. Rebleeding within 30 days occurred in 4 of 14 cases in the direct clipping group and 7 of 32 in the indirect clipping group. The rebleeding rate within 30 days after first-line treatment did not differ significantly among direct clipping group (28.57%) and indirect clipping group (21.88%; *P* = .713) (Table 5).

**Table 4:** Comparison of clinical outcomes between EH-success and EH-failure.

	EH-success (n = 46)	EH-failure (n = 80)	*P*
Rebleeding rate within 30 d	11 (23.91%)	18 (22.50%)	.856^*^
Hospital stay (d)	9 [6–12]	8 [6–11]	.658^‡^
Blood transfusion units	0 [0–2]	0 [0–2]	.762^‡^
Need for surgery	2 (4.35%)	1 (1.25%)	.553^†^

Values are presented as number (%) or median [IQR].

EH = endoscopic hemostasis, IQR = interquartile range.

* Chi-square test.

† Fisher’s exact test.

‡ Mann–Whitney *U* test.

**Table 5:** Comparison of clinical outcomes between endoscopic direct and indirect clipping hemostasis.

	Direct (n = 14)	Indirect (n = 32)	*P*
Rebleeding rate within 30 d	4 (28.57%)	7 (21.88%)	.713^*^
Hospital stay (d)	11 [6–15]	8 [6–10]	.137^†^
Blood transfusion units	0 [0–1]	0 [0–2]	.673^†^
Need for surgery	0 (0%)	2 (6.25%)	1.000^*^

Values are presented as number (%) or median [IQR].

IQR = interquartile range.

* Fisher’s exact test.

† Mann–Whitney *U* test.

**Figure 3.**
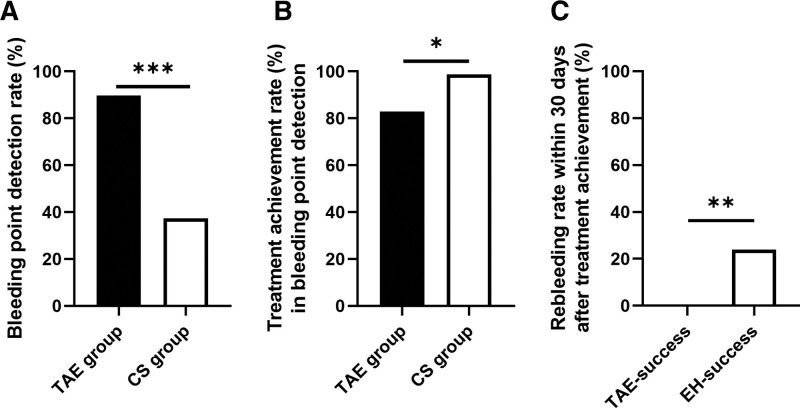
(A) Bleeding point detection rate in the TAE and CS groups. Bleeding point detection rate was higher in the TAE group (89.74%, 35/39) than in the CS group (37.30%, 47/126; *P* < .0001). (B) Treatment achievement rate in cases with a bleeding point detected in the TAE and CS groups. Treatment achievement rate in cases with a bleeding point detected was lower in the TAE group (82.86%, 29/35) than in the CS group (97.87%, 46/47; *P* = .038). (C) Rebleeding rate within 30 d after treatment achievement. Rebleeding rate within 30 d after treatment achievement was significantly lower in TAE-success (0%, 0/29) than in EH-success (23.91%, 11/46; *P* = .005). CS = colonoscopy, CDB = colonic diverticular bleeding, EH = endoscopic hemostasis, TAE = transcatheter arterial embolization. *P* values were determined using chi-square test and Fisher’s exact test.

We analyzed clinical outcomes in bleeding point detection. The rate of bleeding point detection was significantly higher in the TAE group (89.74%) than in the CS group (37.30%; *P* < .0001) (Fig. 3A). Treatment achievement rate in cases of bleeding point detection was lower in the TAE group (82.86%) than in the CS group (98.78%; *P* = .038) (Fig. 3B). Rebleeding rate within 30 days after treatment achievement was significantly lower in the TAE group (0%) than in the CS group (23.91%; *P* = .005) (Fig. 3C).

### 3.5. Complications during TAE

No severe complications of Grade 3 or more were observed during the post-procedural hospitalization period in any patients with TAE. Three cases (four events) with mild complications less than Grade 3 were seen in the 29 CDB cases with TAE, comprising fever >37.5°C in 1 event, abdominal pain with tenderness in 2 events, and colonic bleeding from an ischemic ulcer due to TAE in 1 event.

## 4. Discussion

In this study, we showed that TAE as the first-line treatment prevented rebleeding after initial treatment for CDB patients with extravasation apparent on CECT. In addition, the rebleeding rate was significantly lower with TAE than with endoscopic clipping hemostasis, even when comparing cases in which treatment was achieved. Further, TAE as the first-line treatment for CDB was shown to be a safe procedure, with no severe adverse events observed after TAE in this study.

To the best of our knowledge, this represents the first study to evaluate the efficacy of TAE as first-line treatment for acute CDB compared with other methods of hemostasis. The development of effective hemostatic methods for CDB is an important clinical challenge. Currently, the standard first-line treatment is colonoscopy within 24 hours of hospital visit, and this method is recommended in guidelines for CDB.^[18]^ However, colonoscopy does not contribute to prevention of rebleeding, even though it contributes to identification of the bleeding source for LGIB patients.^[12]^ Our study also demonstrated that endoscopic hemostasis was not associated with a decrease in the risk of rebleeding within 30 days. In daily clinical practice, CECT is increasingly being used as an initial diagnostic modality for acute LGIB patients. According to the previous study, the accuracy of diagnosing the bleeding site was higher with CECT than with colonoscopy.^[8]^ CECT is thus considered as an optimal initial diagnostic modality for detecting the bleeding site in LGIB patients. The present results will help decision-making in line with the diagnostic flow in real-world clinical practice for CDB patients, because we enrolled patients with extravasation apparent on CECT as a first-step examination for the management of CDB. This study demonstrated a low rebleeding rate (7.69%) from TAE as the first-line treatment for CDB with extravasation on CECT in an intention-to-treat analysis (Table 2). Even when comparing cases with treatment achievement between TAE-success and EH-success subgroups (as-treated analysis), TAE showed a lower rebleeding rate than successful endoscopic hemostasis (Fig. 3C). In addition, the rebleeding rate of TAE was significantly lower than that of conservative treatment in the EH-failure subgroup. TAE can thus be considered the most effective hemostatic method in terms of preventing rebleeding in CDB with extravasation apparent on CECT among TAE, colonoscopy, and conservative treatment.

Based on the analysis of adverse events after initial treatment, we confirmed TAE as an acceptably safe first-line hemostatic treatment for acute CDB patients. In this study, no patients experienced severe AEs of Grade 3 or more or mortality after TAE. In 2005, Silver et al reported that the mortality rate following TAE for LGIB was 55% (6/11), while 64% (7/11) of embolized patients had gastrointestinal ischemia.^[19]^ However, recent studies have shown that the incidence of serious ischemic complications of TAE for LGIB was very low (0–5.2%).^[14,15]^ Such improvements in the incidence of AEs depend largely on technical advances such as the development of guide wires and catheter systems that enable super-selective embolization in TAE. Super-selective embolization of the nearest arcade of the vasa recta of the segmental branch is important to prevent TAE-related AEs, including intestinal ischemia. In our study, super-selective embolization was performed in all TAE cases, contributing to both the high rate of treatment success and the low incidence of TAE-related AEs.

Although TAE was superior to colonoscopy as the first-line treatment for CDB with extravasation on CECT, colonoscopy still offers some benefits in the management of CDB. As mentioned above, colonoscopy within 24 hours of a hospital visit improves the rate of bleeding source detection among LGIB patients.^[7]^ Colonoscopy within 24 hours is therefore a therapeutic option for patients without extravasation on CECT or patients who cannot undergo CECT because of impaired renal function or allergy to iodinated contrast media. Another advantage of colonoscopy is that endoscopic hemostasis can be performed if the bleeding point is detected in colonoscopy. Once a bleeding point was detected on colonoscopy, a high rate of treatment achievement (97.87%) was observed in this study (Fig. 3B). However, even in those cases in which a bleeding point was detected, the rebleeding rate was significantly higher in the CS group than in the TAE group (Fig. 3C). Although endoscopic hemostasis did not contribute to prevention of rebleeding, the development of new endoscopic hemostatic methods may lead to improvements in treatment outcomes. The medical cost of TAE (231,100 JPY) is higher than that of endoscopic hemostasis (103,900 JPY). However, CS as the first-line treatment for CDB has higher rebleeding rate than TAE, that leads to higher medical cost associated with additional treatment for rebleeding after first-line treatment.

Several limitations in this study need to be considered. First, this investigation used a retrospective design, obtaining data from the CDB databases of three hospitals. Periods of observation differed between the TAE group and CS group, because the time in which the strategy for managing CDB was used was confirmed to differ between hospitals. In NCU (TAE group), TAE has been performed as the standard first-line treatment for CDB with extravasation on CECT since 2010. On the other hand, in AMU and K hospital (CS group), colonoscopy has been performed as the standard first-line treatment for CDB with extravasation on CECT only since 2016. However, we consider that this difference had little impact on the outcomes, because more recent cases were included in the CS group than in the TAE group. In addition, collecting TAE cases between June 2016 and July 2021 reduces the sample size for TAE as the first-line treatment for CDB and reduces the statistical power to detect a true effect. Second, outcomes of TAE were obtained from only a single center. However, several researchers have demonstrated the efficacy and safety of TAE for LGIB. We therefore believe that our results for TAE as first-line treatment for CDB can be generalized to institutions employing specialists in interventional radiology. Third, the endoscopic hemostatic method used in this study was clipping alone. Recently, endoscopic band ligation has been reported as an effective treatment for CDB.^[20–23]^ However, a recent systematic review and meta-analysis demonstrated no significant differences in rates of initial hemostasis or rebleeding within 30 days.^[24]^ Because endoscopic clipping is widely used for the initial treatment of CDB, we consider that the outcomes of TAE compared with endoscopic clipping hemostasis reflect real world clinical practice. Fourth, the majority of CDB patients are extravasation-negative on CECT. In our study, the population of CDB showing extravasation on CECT represented 25.7% (188/730) of patients who underwent CECT on admission. Fifth, the long-term outcome is still unclear because most of the patients was not followed-up for >30 days. In this study, in the TAE group, there was no rebleeding case >5 days after initial treatment. However, in the CS group, 5 of 29 (17.2%) rebleeding cases occurred >5 days after initial treatment. These results suggest that once hemostasis is achieved, the hemostatic effect of TAE might persist over a long-term period. To solve these issues, prospective studies are needed to elucidate the optimal initial treatment for CDB.

In conclusion, we showed that TAE is an effective and safe hemostatic method for first-line treatment of CDB showing extravasation on CECT. TAE is a useful alternative to endoscopic hemostasis for first-line treatment of CDB.

## Acknowledgments

We are grateful to all physicians who treated CDB patients in the present study.

## Author contributions

All authors had full access to all of the data in the study and Dr Katano takes responsibility for the integrity of the data and the accuracy of the data analysis. All authors confirm that they have read and approved the final version of the manuscript and agree to its publication.

**Conceptualization:** Yuki Kojima, Takahito Katano.

**Data curation:** Yuki Kojima, Takahito Katano.

**Formal analysis:** Yuki Kojima, Takahito Katano.

**Investigation:** Yuki Kojima, Takahito Katano, Takaya Shimura, Masashi Shimohira, Tomoya Sugiyama, Masahide Ebi, Takahito Harada, Yuki Yamamoto, Yoshikazu Hirata, Hiromi Kataoka.

**Methodology:** Yuki Kojima, Takahito Katano.

**Project administration:** Yuki Kojima, Takahito Katano.

**Resources:** Yuki Kojima, Takahito Katano, Takaya Shimura, Masashi Shimohira, Tomoya Sugiyama, Masahide Ebi, Takahito Harada, Yuki Yamamoto, Yoshikazu Hirata, Hiromi Kataoka.

**Software:** Takahito Katano.

**Supervision:** Hiromi Kataoka.

**Validation:** Yuki Kojima, Takahito Katano.

**Visualization:** Yuki Kojima, Takahito Katano.

**Writing – original draft:** Yuki Kojima.

**Writing – review & editing:** Takahito Katano.
